# Lineage-restricted sympathoadrenal progenitors confer neuroblastoma origin and its tumorigenicity

**DOI:** 10.18632/oncotarget.27636

**Published:** 2020-06-16

**Authors:** Chia-Lung Yang, André Serra-Roma, Marco Gualandi, Nicole Bodmer, Felix Niggli, Johannes Hubertus Schulte, Peter Karl Bode, Olga Shakhova

**Affiliations:** ^1^ Department of Medical Oncology and Hematology, University Hospital Zürich, Zürich, Switzerland; ^2^ Department of Oncology, Children Hospital of Zürich, Zürich, Switzerland; ^3^ Department of Pediatric Hematology, Oncology and SCT, Charité University Hospital, Berlin, Germany; ^4^ Department of Surgical Pathology, University Hospital Zürich, Zürich, Switzerland

**Keywords:** neuroblastoma, neural crest stem cells, sox geness

## Abstract

Neuroblastoma (NB) is the most common cancer in infants and it accounts for six percent of all pediatric malignancies. There are several hypotheses proposed on the origins of NB. While there is little genetic evidence to support this, the prevailing model is that NB originates from neural crest stem cells (NCSCs). Utilizing *in vivo* mouse models, we demonstrate that targeting *MYCN* oncogene to NCSCs causes perinatal lethality. During sympathoadrenal (SA) lineage development, SOX transcriptional factors drive the transition from NCSCs to lineage-specific progenitors, characterized by the sequential activation of *Sox9/Sox10/Sox4/Sox11* genes. We find the NCSCs factor SOX10 is not expressed in neuroblasts, but rather restricted to the Schwannian stroma and is associated with a good prognosis. On the other hand, SOX9 expression in NB cells was associated with several key biological processes including migration, invasion and differentiation. Moreover, manipulating *SOX9* gene predominantly affects lineage-restricted SA progenitors. Our findings highlight a unique molecular SOX signature associated with NB that is highly reminiscent of SA progenitor transcriptional program during embryonic development, providing novel insights into NB pathobiology. In summary, we provide multiple lines of evidence suggesting that multipotent NCSCs do not contribute to NB initiation and maintenance.

## INTRODUCTION

Neuroblastoma (NB) is a pediatric malignancy affecting infants and young children with median age at diagnosis is 17 months [1]. Multimodality treatment of high-risk NB can be effective, but up to fifty percent of children experience recurrent disease [2–4]. Despite various sequencing efforts to systemize and decipher its complexity, the biology of NB has remained largely elusive. The remarkable clinical and biological diversity of NB can vary from patient to patient and one of the hallmarks of NB is its unique capacity to undergo spontaneous regression [5]. Such composite nature of NB cannot be explained genetically since high-throughput sequencing uncovered low mutational burden in primary NB [6].

Several recent studies presented convincing evidence that *in vitro* NCSCs can act as a cellular population capable of NB initiation, when transduced with *MYCN*, *ALK*^F1174L^ and *ALK*^R1275Q^ oncogenes and injected into immunocompromised mice [7–9]. These findings and other notable parallels shared by NCSCs and NB cells, including remarkable capacity to metastasize, have led to a number of recent reviews proposing NCSCs as the cell-of-origin and CSCs in NB [10–13]. However, despite a growing number of reports, no definitive evidence has been presented to whether NCSCs can initiate NB *in vivo* in mouse models and therefore, serve as a candidate cellular population as the cell-of-origin of NB. Moreover, no functional evidence has been obtained for the presence of NCSCs in human patient’s biopsies.

Here, by combining *in vivo* mouse genetic approach and the functional analysis of human NB cell lines, we present multiple lines of evidence that multipotent NCSCs do not contribute to NB initiation and maintenance. Comprehensive analysis of the signature NCSCs stemness genes, SOX10 and SOX9, in combination with the induction of the neural crest (NC) program by overexpression of SOX9, uncovered the lack of NCSCs within human biopsies. Notably, the genetic profile of NB cells shared a striking resemblance to the embryonic lineage-restricted progenitors expressing *SOX4*/*SOX11* genes. Taken together, our study provides novel insights into our understanding of NB biology, and lays the foundation for better targeted therapeutic strategies.

## RESULTS

### Targeting the MYCN oncogene to the neural crest stem cells *in vivo* results in perinatal lethality

**Figure 1:**
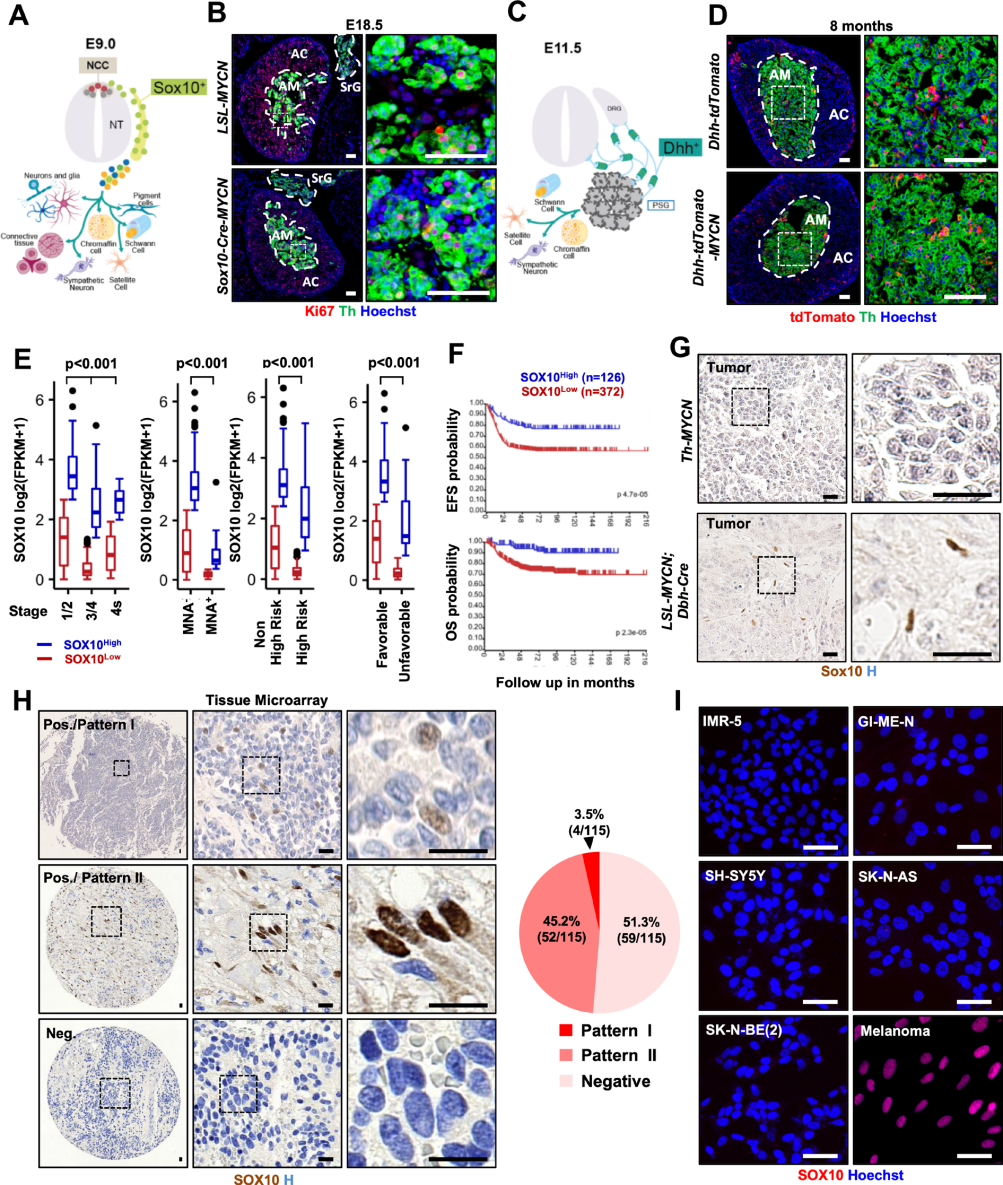
Targeting MYCN to Sox10+ NCSCs does not lead to NB formation and SOX10 is predominantly expressed in Schwannian stromal compartment. (**A**, **C**) Neural crest cells and their progeny at E9 (A) and E11.5 (C). (**B**) Immunostaining for Ki67 and Th in adrenal gland (AG) and suprarenal ganglia (SrG) from *LSL-MYCN*, and *Sox10-Cre; LSL-MYCN* at E18.5. Scale bars: 50 μm. (**D**) Immunostaining for Th in AG from *Dhh-Cre; LSL-tdTomato*, and *Dhh-Cre; LSL-tdTomato; LSL-MYCN* mouse. Scale bars: 50 μm. (**E**) Differential *SOX10* expression in neuroblastoma (INSS stages (1/2, *n =* 199; 3/4, *n =* 246; 4s, *n =* 53), *MYCN* status (MNA^-^, *n =* 401; MNA^+^, *n =* 92) risk group (non-high, *n =* 322, high, *n =* 176) and response to treatment (favorable, *n =* 181; unfavorable, *n =* 91)) in cohort 1. Statistics analyzed by Kruskal-Wallis or Mann-Whitney test. (**F**) Kaplan-Meier curve of event-free survival (EFS) and overall survival (OS) of neuroblastoma patients with high and low *SOX10* gene expression. (**G**) Sox10 staining in tumor from *Th-MYCN* and *LSL-MYCN; Dbh-iCre* mice. Scale bars: 25 μm. (**H**) Immunohistochemistry for SOX10 in human neuroblastoma tissue microarray. Pie chart indicates percentage and case numbers. Scale bars: 20 μm (**I**) Immunofluorescence for SOX10 in neuroblastoma cell lines. Positive control: M111031 melanoma cell line. Scale bars: 50 μm NCC: neural crest cell, NT: neural tube, NC: notochord, DA: dorsal aorta, DRG: dorsal root ganglia, PSG: primary sympathetic ganglia, AG: adrenal gland.

Previous reports suggested that oncogenic transformation of NCSCs can initiate NB formation *in vitro* [7–9, 14]. These observations prompted us to investigate whether multipotent NCSCs can also serve as NB cell of origin *in vivo.* Since *MYCN* amplification is the most common genetic aberration in sporadic NB and occurs in approximately 25 percent of tumors [2], we used LSL-*MYCN* mice, a genetically engineered mouse model in which *MYCN* oncogene is activated upon *Cre*-mediated recombination [14]. To induce the expression of *MYCN* in NCSCs, we took advantage of *Sox10-Cre* line, which allows *Cre*-mediated recombination from E9 onwards (Figure 1A) [15]. The absence of mice carrying both transgenes at postnatal day 21 (several independent litters were analyzed, total *n =* 79 mice; Supplementary Figure 1A) suggested the presence of embryonic or perinatal lethality. As shown in Supplementary Figure 1A, PCR analysis revealed the presence of wild-type (*WT*) genotype in 12/79 mice, *Cre* genotype was detected in 28/79 mice, LSL*-MYCN* genotype was present in 39/79 mice and none of the genotyped animals carried both transgenes (0/79). In order to investigate whether LSL-*MYCN; Sox10-Cre* mice were born alive, we genotyped entire litters at postnatal day 0. To increase the chance of pups carrying both transgenes, we crossed mice homozygous for LSL*-MYCN* allele with *Sox10-Cre* mice as shown in Supplementary Figure 1B. Based on our breeding scheme, we expected that 100% of resulting offspring should be hemizygous for LSL*-MYCN* allele, and 50% should inherit *Cre* transgene resulting in approximately 50% of pups carrying both transgenes. We have analyzed 16 pups at postnatal day 0 and observed the expected genetic distribution, 16/16 pups inherited LSL*-MYCN* allele, 6/16 pups in addition to the LSL*-MYCN* allele also carried *Cre* transgene (Supplementary Figure 1B). These data reveal that pups carrying LSL-*MYCN; Sox10-Cre* genotype are born at Mendelian ratio. However, all double transgenic LSL-*MYCN; Sox10-Cre* pups (*n =* 6) did not move after birth suggesting that despite developing to full term, these pups were likely stillborn. The possible mechanisms underlying this lethality might include the presence of a respiratory failure in these pups due to severe defects in sympathetic nervous system as well as abnormalities in other tissues and organs.

To circumvent perinatal lethality, we time-mated LSL-*MYCN* and *Sox10-Cre* mice and isolated embryos at embryonic day 18.5 (E18.5). Gross anatomical examination of LSL-*MYCN; Sox10-Cre* embryos did not reveal any significant abnormalities (Supplementary Figure 1C). To assess whether NB formed during prenatal development, we analyzed sympathoadrenal (SA) progenitors. Immunofluorescence revealed that at E18.5, SA progenitors in the adrenal medulla (AM) did not exhibit an increased proliferation as judged by anti-Th (tyrosinase hydroxylase) and proliferation marker Ki67 (Figure 1B and Supplementary Figure 1D). Of note, SA progenitors in the suprarenal ganglia (SrG) proliferated at a similar rate in both control and LSL-*MYCN; Sox10-Cre* embryos (Figure 1B). While NB tumors can develop anywhere in the sympathetic nervous system, the most prevalent anatomical location is AM (40% of localized NB and up to 60% of widespread disease). Nevertheless, we cannot rule out the possibility that NB might have originated in other sympathetic ganglia (SG) of LSL-*MYCN; Sox10-Cre* embryos. However, taking into account the absence of an increased proliferation and morphological abnormalities within AG and SrG, it seems to be an unlikely scenario.

Interestingly, another NB oncogene, *ALK*^F1174L^, when targeted to NCSCs using *Sox10-Cre*, also leads to lethality albeit at the earlier embryonic time-point [16].

Similarly to Schwann cell precursors (SCPs), *Desert Hedgehog* (Dhh)-expressing cells can give rise to a subpopulation of chromaffin cells in the AM, and therefore, can potentially be the cell-of-origin of NB *in vivo* (unpublished data) [22, 23]. To address this possibility, we crossed LSL-*MYCN* mice with *Dhh-Cre* [24] transgenic line, which drives *Cre* expression from E12 onwards (Figure 1C). LSL-*MYCN; Dhh-Cre* mice were born at expected Mendelian ratio, viable and appeared morphologically normal. To identify the presence of *MYCN* overexpression, we used the *luciferase* reporter and performed an *in vivo* imaging system (IVIS) to monitor the *MYCN*-positive cells in the sympathetic chain and in the AM. Despite the successful induction of *MYCN* oncogene in *Dhh*-expressing cells we did not observe NB formation (mice were aged up to 8 months, Figure 1D). It is important to note, however, that our data cannot exclude that a population of SCPs molecularly distinct from Dhh-expressing cells still could be a candidate NB cell-of-origin population.

In order to exclude a potential toxicity of the LSL-*MYCN* transgene, we crossed LSL-*MYCN* mice with SA progenitor-specific *Dbh-iCre* mice and observed NB development as previously reported (Supplementary Figure 1E, 1F) [14].

Taken together, our *in vivo* data demonstrate that only SA progenitors are capable of tumor initiation in the context of *MYCN* overexpression in the transgenic mice.

### Human neuroblastoma samples contain SOX10-expressing cells but its expression is predominantly found in Schwann cells

A leading hypothesis posits that NB occurs as a result of the deregulation of NC development via hijacking pathways controlling NC stemness [11, 12, 25]. Stem cell/ progenitor identity is determined by lineage-specific transcription factors. To test whether NB cells express NCSCs lineage genes, we analyzed the expression of a master regulator of NCSCs identity, transcription factor SOX10 [26–28]. Our data reveal that SOX10 was expressed in human NB samples and was associated with a good prognosis (Figure 1E, 1F, *n =* 498, *P* < 0.001 and Supplementary Figure 2A–2C). This result is somewhat counterintuitive, because the presence of NCSCs-like cells would rather be associated with more aggressive tumor behavior. Indeed, in melanoma, another NC-derived malignancy, *SOX10* expression predisposes to tumor development and is associated with a bad prognosis [29]. In order to investigate SOX10 expression on a cellular level, we have performed immunohistochemical analysis of NB tissue derived from two independent genetically engineered mouse models (*TH-MYCN* [30] and LSL-*MYCN*; *Dbh-iCre* [14]). Strikingly, SOX10 expression was restricted to Schwann cells and absent from tumor cells (Figure 1G). Delving deeper, we performed immunostaining for SOX10 in 115 human NB samples (Figure 1H). In human NB tissue, *SOX10* expression pattern could be divided into neuroblasts-specific (hereinafter referred to as “Pattern I”) 3.5% of NB (4 patients); Schwannian stroma-restricted (hereinafter referred to as “Pattern II”) 45.2% (52 patients) and SOX10-negative, 51.3% (59 patients) (Figure 1H and Supplementary Figure 2D). Human NB tissue is composed of two spatially juxtaposed cell types, neuroblasts and Schwann cells, both cell types derive from NC lineage. This tissue composition mimics normal embryonic development in particular resembling the architecture of SG including AM at the late stage of NC development. It is widely accepted that Schwannian stroma as well as intratumoral Schwann cells in NB derive from non-neoplastic cell rather than from a common neoplastic progenitor. Taking into account that SOX10 is expressed in the glial cells in the peripheral nervous system during normal embryonic development, it is not surprising to observe its expression in NB Schwannian stroma. Moreover, the absence of SOX10 expression in tumorigenic neuroblasts in human NB tissue is in agreement with the lack of Sox10 expression in neurons of peripheral nervous system [27]. Since Schwannian stroma-rich NBs are defined as clinically nonaggressive, it explains why SOX10^HIGH^ tumors are associated with a good prognosis. Taken together, our data reveal that NB tissue is largely devoid of the presence of cells with neural crest characteristics as demonstrated by the absence of SOX10 expression in tumorigenic compartment of NB.

Finally, we have assessed a panel of previously established human NB cell lines for the expression of SOX10 (Figure 1I). We examined the presence of SOX10^+^ cells in 15 different human NB cell lines and show that none of human NB cell lines revealed the presence of SOX10^+^ cells neither by Western blotting nor by immunostaining and RNA sequencing (Figure 1I and Supplementary Figure 2E, 2F). These observations are in agreement with the fact that NB cell lines entirely lack Schwannian stroma component and are primarily composed of neuroblasts.

The lack of SOX10 expression in tumorigenic compartment of NB argues against NCSC-based genetic program underlying NB pathogenesis. Because SOX10 is a “master regulator” and belongs to the top of the regulatory transcriptional hierarchy controlling NCSC identity and fate, it is very unlikely that another population of SOX10-neagtive NCSCs is present within NB.

### The SOX9-driven neural crest induction does not initiate NC signature in human NB cell lines

**Figure 2:**
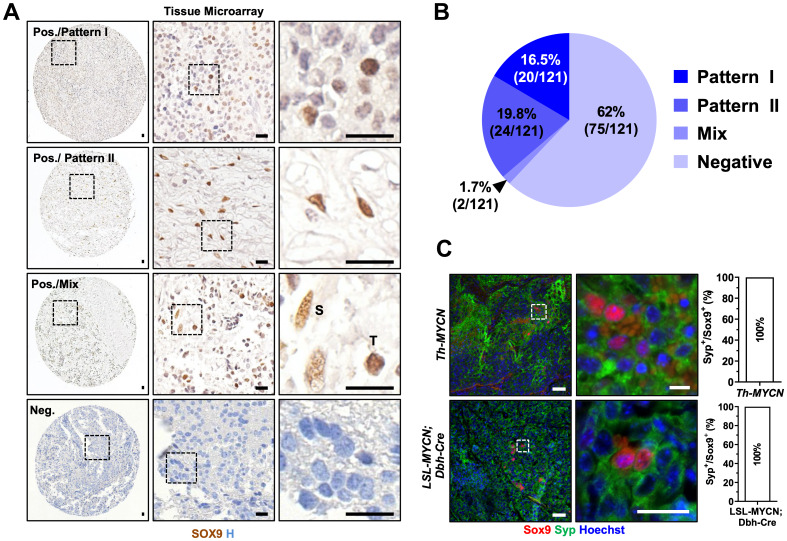
SOX9 is expressed in tumorigenic neuroblasts in human and mouse neuroblastoma samples. (**A**) Immunohistochemistry for SOX9 in human neuroblastoma tissue microarray. Scale bars: 20 μm. (**B**) Pie chart indicates percentage and case numbers from the tissue microarray represented in (A). (**C**) Immunofluorescence for Sox9 and Syp in tumor from *Th-MYCN* and from *LSL-MYCN; DBH-Cre* mice. Scale bar: 50 μm.

Sox9, another member of SoxE family of transcription factors, plays a crucial role in NC development [31]. It has been recently suggested by Kanduri and colleagues that SOX9 plays a key role in stem cell proliferation in human NB [33]. We have studied SOX9 expression in a large cohort of human NB samples and observed that its expression was correlated with unfavorable prognosis (Supplementary Figure 3A–3E) (*n =* 498). While SOX9 expression did not differ between different stages of NB, its expression was increased in *MYCN* amplified tumors (*p <* 0.01), as well as in unfavorable samples as compared to samples with favorable prognosis (*p <* 0.05). Immunohistochemistry for SOX9 protein revealed that 16.5% of all samples (20/121) exhibited “Pattern I” (neuroblast-restricted) and 19.8% (24/121) showed “Pattern II” (Schwannian stroma-restricted), respectively (Figure 2A, 2B). However, the majority of samples (62%;75/121) were devoid of SOX9^+^ cells. Next, we investigated the expression of SOX9 in human NB cell lines at the RNA as well as at the protein level as shown in Supplementary Figure 4A, 4B. In striking contrast to SOX10, the expression of SOX9 was readily detected in the majority of human NB cell lines (Supplementary Figure 4A) indicating that SOX9 is present in neuroblasts. Subsequently, we investigated mouse NB tissue derived from *TH-MYCN* and LSL-*MYCN*; *Dbh-iCre* mice. We observed Sox9^+^ cells and they morphologically resembled neoplastic neuroblasts (Supplementary Figure 4C, 4D, upper panels). Both mouse tumors appeared stroma-poor, so it might explain the lack of Sox9^+^ Schwann cells. Interestingly, no Sox9^+^ cells were present before the onset of tumor initiation as shown in Supplementary Figure 4C, 4D (lower panels). The fact that non-neoplastic tissue was devoid of Sox9-expressing cells points towards a lack of *MYCN*-driven regulation of Sox9 expression. To test whether Sox9^+^ cells were present in the human AM, we have performed immunohistochemical analysis for SOX9 protein in the tissue at the gestational weeks 14 and 17. As shown in Supplementary Figure 4E, no SOX9-expressing cells were detected in human fetal AM. These data are in accordance with previously published reports demonstrating that AM is completely devoid of SOX9^+^ cells during embryonic development [28]. To verify our findings also in the non-transgenic *wild-type* mouse tissue of AM, we took the advantage of Sox9 reporter mouse strain [32], a line in which GFP expression is driven by the endogenous *Sox9* promoter. AM did not contain any GFP^+^ cells, while they could readily be detected in the gut (Supplementary Figure 4F, 4G).

To validate the identity of Sox9^+^ cells observed in mouse NB tissue, we have performed the immunostaining for synaptophysin (SYP), a neuronal/neuroendocrine lineage marker, and virtually all SOX9^+^ cells showed strong SYP positivity in mouse neoplastic neuroblasts (100% respectively, Figure 2C). As mentioned earlier, both mouse tumors were stroma-poor and this observation could explain the lack of Sox9^+^ Schwann cells.

**Figure 3:**
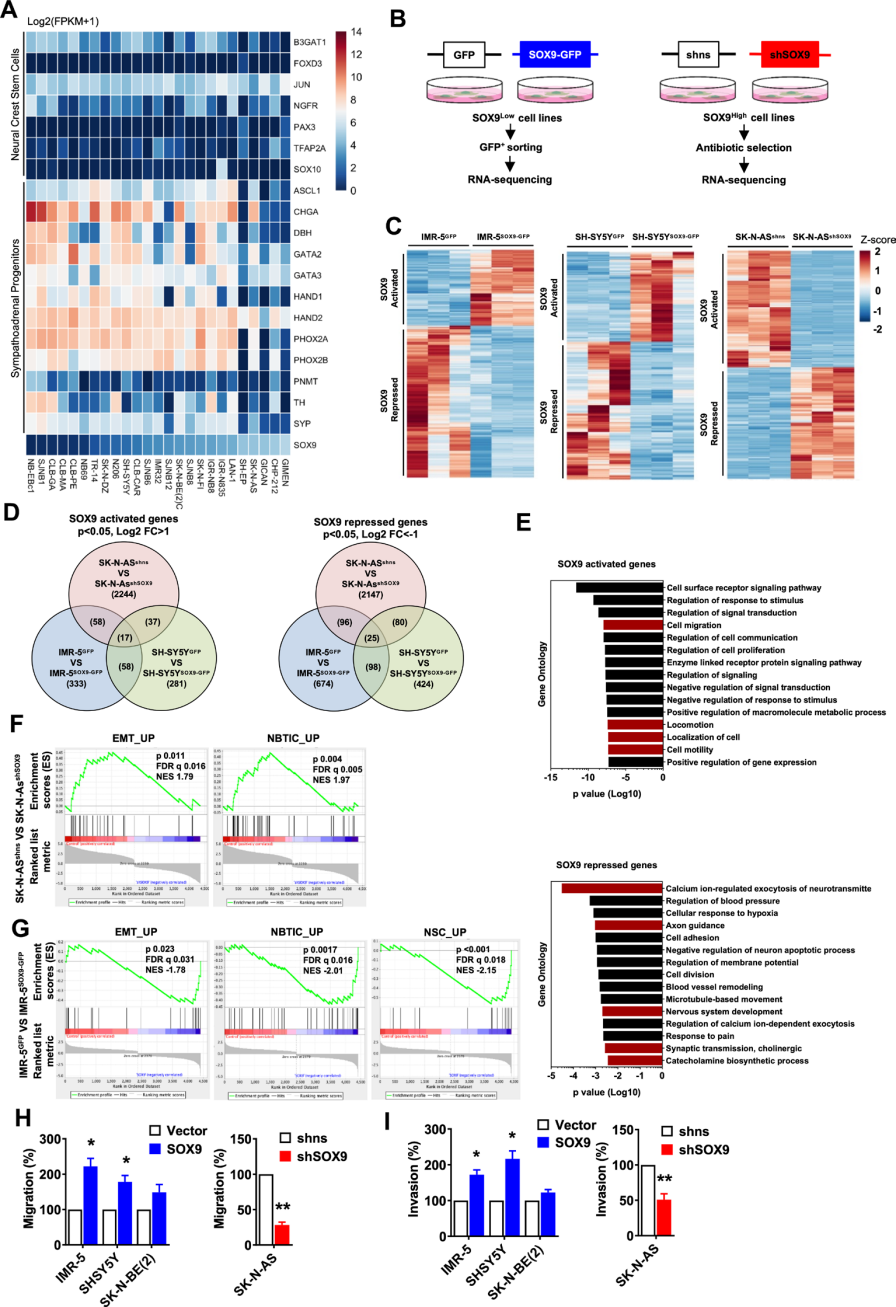
Oncogenic properties of SOX9 in neuroblastoma are associated with SA lineage progenitors. (**A**) Differential expression of NCSC and SA-progenitor signature genes in neuroblastoma cell lines from database 1. (**B**) Experimental procedure of harvesting pure transfected cells for RNA-sequencing. *n =* 3 independent samples were subjected to RNA sequencing. (**C**) Genes differentially expressed in IMR-5^vetor^ versus IMR-5^SOX9^, SH-SY5Y^vetor^ versus SH-SY5Y^SOX9^, and SK-N-AS^shns^ versus SK-N-AS^shSOX9^ cells. (**D**) Venn diagram shows overlap of SOX9 activated and repressed genes from three RNA sequencing datasets individually. For SOX9 activated genes, only genes with significant *p* value and log2 fold change higher than one are selected. For SOX9 repressed genes, only genes with significant p value and log2 fold change less than minus one are selected. Overlapping gene numbers are indicated. (**E**) Gene ontology analysis of SOX9 activated and repressed genes commonly overlapping in either two or three datasets. Top fifteen GO results base on *p* value are shown. Bar graph shows SOX9 activated genes are involved in cell migration related categories (red), and SOX9 repressed genes are associated in neuron differentiation (red). (**F**, **G**) Migration (F) and invasion (G) assays of SOX9 overexpressed and knockdown cells. Scale bar: 50 μm. Results in (F, G) are shown as mean ± SEM and statistics are analyzed by Student’s t-test.

Taken together these data do not seem to support the stem cell identity of SOX9-expressing cells but rather indicate that SOX9^+^ cells might represent SA lineage-restricted cell population. To test this hypothesis, we further investigated the molecular signature of SOX9^+^ cells. The analysis of a publicly available database of NB cell lines revealed that SOX9 expression was clearly associated with the expression of SA lineage markers such as PHOX2A, PHOX2B, DBH and TH (Figure 3A). Moreover, no NC stem cell signature was observed in SOX9-expressing NB cell lines (Figure 3A).

Given its crucial role in the step of NC induction during development [34] and observations by Mondal et al., we hypothesized that SOX9 overexpression could suffice to induce *de-novo* stemness in NB cells. To test this hypothesis, loss- and gain-of-function studies were performed to manipulate the levels of SOX9 in established NB cell lines. Cell lines with low level of endogenous SOX9 expression (IMR-5, SHSY5Y, SK-N-BE (2)) were used to induce *SOX9* overexpression and cell lines with high level of SOX9 expression (SK-N-AS, CHP-100) were used to perform *SOX9* KD (schematically illustrated in Figure 3B). We next confirmed both overexpression and knock-down at the protein level as shown in Supplementary Figure 5A, 5B. Since both vectors (*SOX9* overexpression and *SOX9* knock-down) carried *GFP* sequence, we have decided to enrich for SOX9-altered cells and performed FACS sorting (Supplementary Figure 5C). Subsequently, we have subjected GFP-sorted cells to RNA sequencing (Figure 3C). We have then analyzed expression profiles of *SOX9* activated vs *SOX9* repressed genes (Figure 3D, 3E) and observed that while the overexpression of *SOX9* altered processes such al cell migration, cell motility and locomotion, knock-down of *SOX9* was associated with changes in axon guidance, nervous system development, synaptic transmission and regulation of exocytosis of neurotransmitters (Figure 3E). To functionally assess the relevance of the genetic changes we observed, we have performed several *in vitro* assays. To address whether *SOX9* overexpression induced EMT-like processes, we have performed *in vitro* migration and invasion assays (Figure 3F, 3G). Our data demonstrated that while overexpression of *SOX9* resulted in an enhanced migration and invasion in IMR-5 and SHSY5Y cells, knock-down of *SOX9* in SK-N-AS was associated with clearly pronounced decrease in migration and invasion (Figure 3F, 3G and Supplementary Figure 5F). We next examined if SOX9 could confer oncogenic properties of NB cells and performed *in vitro* colony formation assay and *in vivo* tumor formation. As shown in Supplementary Figure 6A, 6B, *SOX9* overexpression increased number of colonies and knock-down of *SOX9* resulted in fewer colonies and also significantly reduced tumor volume. To tests whether these oncogenic properties were associated with increased stemness, we have assessed sphere-forming capacities of SOX9-altered cells. As shown in Supplementary Figure 6C, overexpression of *SOX9* did enhance sphere formation in IMR-5, SH-SY5Y and SK-N-BE (2) and reduced it in SK-N-AS. This result might seem counterintuitive since our previous data indicated that SOX9^+^ cells represent committed progenitors rather than undifferentiated stem cells. However, it is important to note that sphere-forming *in vitro* assay on its own is not sufficient to provide accurate readout of the number of stem cells. Moreover, a majority of our NB cultures failed to generate spheres from a single cell and therefore, we have decided to perform this assay in high-density cultures. This *per se* might have jeopardize the purity of such an assay and therefore, we cannot exclude that sphere fusion could influence our results.

As we have shown earlier, our RNA sequencing data suggested a possible role of SOX9 in neuronal differentiation. First, we have quantified the number of TH-positive cells upon *SOX9* overexpression (Supplementary Figure 6D). In two independent NB cell lines, SH-SY5Y and SK-N-BE (2), SOX9-overexpressing cells were characterized by a strong reduction in TH-positivity indicating significant degree of de-differentiation. To functionally assess whether SOX9 plays a role in neuronal differentiation, we have performed retinoic acid (RA)-induced *in vitro* differentiation (Supplementary Figure 6E, 6F). As illustrated in Supplementary Figure 6E, cells overexpressing SOX9 were exposed to RA for 96 hours and subjected to gene expression analysis for selected genes. RT-PCR revealed that while in control cells RA exposure induced expression of DBH (dopamine beta-hydroxylase), NSE (neuron-specific enolase), SLC6A2 (norepinephrine transporter gene) and SYP (synaptophysin), SOX9 overexpression appeared to interfere with the induction of expression of these genes. These data indicate that an overexpression of SOX9 (in this particular case SOX9 overexpression level was nearly 100 times higher compared to control cells) can prevent RA-induced neuronal differentiation.

### SOX neuroblastoma signature is highly reminiscent of sympathoadrenal progenitor program during embryonic development

To this end, we have identified SOX9 expression in neoplastic neuroblasts, characterized by the presence of lineage-specific markers such as synaptophysin. We also showed that interfering with *SOX9* gene revealed its role in migration, invasion and differentiation. Moreover, our data demonstrate that SOX9 can also confer oncogenic properties of NB cells. However, all of these effects seem to be restricted to the lineage-committed SA progenitors.

**Figure 4:**
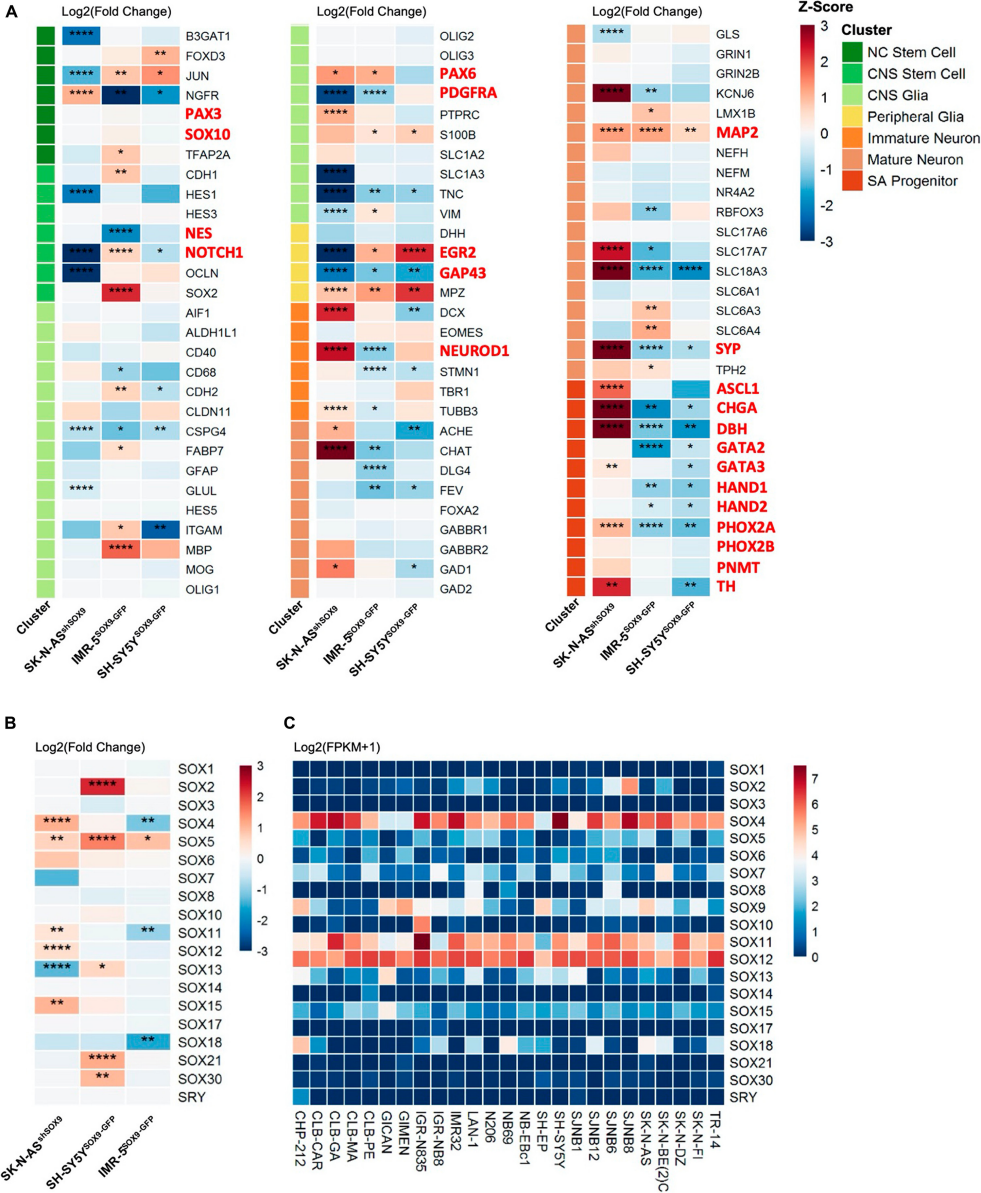
Lack of NC induction upon SOX9 overexpression and SOX molecular signature associated with NB. (**A**) Expression fold change of NC and CNS stem cell, CNS and Peripheral Glia, Immature and Mature Neurons, and SA Progenitor genes in IMR-5^SOX9vsVector^, SH-SY5Y^SOX9vsVector^ and SK-N-AS^shSOX9vsVector^. (**B**) Expression fold change of *SOX* family genes in IMR-5^SOX9vsVector^, SH-SY5Y^SOX9vsVector^ and SK-N-AS^shSOX9vsVector^ (^*^ *p <* 0.05; ^**^ *p <* 0.01; ^***^ *p <* 0.001; ^****^ *p <* 0.0001). (**C**) SOX family gene expression in neuroblastoma cell lines (*n =* 25).

To delve deeper into molecular signature of SOX9 cells, we have dissected our RNA sequencing data with respect to genes specific for stem cell/progenitor/differentiated cells stages. As shown in Figure 4A, several clusters were revealed including gene signature specific for NC stem cells, central nervous system (CNS) stem cells, CNS glial cells, peripheral glia, immature neuron/progenitor, mature neurons and SA progenitors. In line with our previous data, SOX10 expression was not changed upon *SOX9* gene changes (Figure 4A). It is in striking contrast to early development, where Sox9 expression is sufficient to induce NC-like phenotype and the expression of Sox10 [34]. On the contrary, NB cells seem not to follow NC scenario and fail to induce SOX10 expression upon SOX9 overexpression.

Given the importance of this key step in NC formation, the lack of similar genetic program *per se* indicates that genetic resemblance between NCSCs and NB cells might be overstated. Among genes in CNS stem cell cluster, we have observed that upon SOX9 overexpression in IMR-5 cells, expression of several key genes including SOX2, Nestin, NOTCH1, PAX6 and PDGFRA has changed. A very interesting study performed by Bronner and colleagues have recently demonstrated that ectopic expression of *MYCN* when targeted to NC in chicken embryos drives towards CNS-like identity concomitant with a lack of normal NC identity [47]. Another striking change in gene expression was associated with genes in SA progenitor cluster including ASCL1, CHGA, DBH, GATA2 and GATA3, HAND1 and HAND2, PHOX2a and PHOX2B as well as PNMT and TH genes (Figure 4A). These changes are suggestive of SOX9-dependent SA lineage maintenance and/or differentiation rather than stemness-associated role.

Intriguingly, we find that the expression of several SOX genes has been modulated upon changes in *SOX9* gene (Figure 4B). Among all *SOX* genes, only two members, namely SOX4 and SOX11, were upregulated upon *SOX9* overexpression and downregulated upon *SOX9* knock-down. These results prompted us to investigate RNA expression of all *SOX* genes in a panel of established NB cell lines. The subsequent analysis of all 21 *SOX* genes in human NB cells demonstrated that three members of SOX C subgroup (SOX4, SOX11 and SOX12) were profoundly expressed (Figure 4C). In line with our previous observations, SOX10 RNA was not detected in any of NB cell lines analyzed (Figure 4C).

Sox4 and Sox11 are known to exert pleiotropic effects in the central nervous system (CNS) and peripheral nervous system (PNS) during embryonic development [35, 36]. They play a role in determining neuronal identity of CNS precursors, as well as driving proliferation and maintaining survival of SA progenitors [35]. The overlapping expression of Sox4 and Sox11 has been shown to be restricted to the immature Phox2b^+^/Th^+^ SA progenitors and both proteins are not expressed in NCSCs [35]. Loss of both proteins does not influence the number of Sox10^+^ NCSCs and glia, further demonstrating that its function is restricted to SA progenitors [35].

The full functional analysis of the possible role of SOX4, SOX11 and SOX12 is well beyond the scope of the current study but such experiments can provide further insight into our understanding of NB pathogenesis.

## DISCUSSION

Despite decades of speculations, there has been no compelling functional evidence supporting the relevance of the suggested link between NCSCs and NB cells. Given the prominence of the CSC theory and its proven role in cancer initiation in a wide range of tissues, many researchers have assumed that NB originates from multipotent NCSCs. Here, we show using *in vivo* mouse models of NB, that multipotent Sox10-expressing NCSCs do not have the capacity to sustain oncogenic activation leading to NB formation. Instead, initiating oncogenic transformation in NCSCs results in perinatal lethality. Interestingly, in humans NB can occur in patients with Hirschsprung disease, a neurocristopathy linked to the impaired function of NCSCs cells [17–21]. These observations corroborate our findings in GEM models and suggest that NB can occur even in the absence of proper functioning of NCSCs.

On the contrary, there has also been controversy regarding the existence of NCSCs within NB tissue. Large scale sequencing data demonstrated the presence of NC-like transcriptional circuitry in NB cell cultures as defined by the expression of AP-1 [37]. NB cell lines are heterogeneous and include three distinct cellular phenotypes, neuroblastic N-type, non-neuronal substrate-adherent S-type and intermediate, I-type cells [38–40]. While N-type cells have been suggested to resemble SA progenitors, S-type cells have been attributed similarities to Schwann cells. Previously it has been proposed that NB I-type cells are NCSCs [41]. Our data, however, demonstrate that NB human cell lines lack the expression of a *bona fide* NCSCs marker, SOX10. Instead, analysis of human biopsies from NB patients revealed the SOX10 expression in restricted to the cells of NB Schwannian stroma. Based on the International Neuroblastoma Pathology Classification (INPC), four distinct diagnostic subgroups include ganglioneuroma, ganglioneuroblastoma (GNB) intermixed, GNB nodular and NB. The first three are characterized as Schwannian stroma-rich and are associated with good prognosis. NB, however, is usually SS-poor and can also be subdivided into undifferentiated, poorly differentiated and differentiated subgroups [42]. Interestingly, our data on the SOX10 expression in the Schwannian stroma is in agreement with the expression and functional role of Sox10 in the peripheral glia during embryogenesis [26].

Given the fact that human NB cell lines were devoid of SOX10 expression, we attempted to initiate the neural crest (NC) program in NB by overexpressing *SOX9* gene, which plays a crucial role in NC induction during embryonic development [34, 43, 44]. We found that SOX9 does not initiate stemness properties in NB cells, though oncogenic properties associated with EMT induction and impaired differentiation were acquired. These data suggest that oncogenic propensities of NB cells including its metastatic potential is mediated differently than in embryonic NCSCs. During embryogenesis, Sox9 expression is not detectable in the SA lineage progenitors in mice [28], which in accordance with our data in mouse and human adrenal grand.

In contrast to embryogenesis, we show that a subset of NB cells displays SOX9-positivity both in mouse NB models and in human NB tissue. All SOX9-positive cells (100%) expressed markers of SA lineage committed progenitors.

Several recent publications have described the NB transcriptional core regulatory circuitry (CRC) [37, 45, 46]. Our data further substantiate the role of CRC in NB initiation and maintenance, highlighting the origin of NB from SA lineage restricted progenitors. In line with our findings, another report suggests that ectopic expression of *MYCN* in NC domain of chicken embryos drives towards CNS-like identity concomitant with a lack of normal NC identity [47]. The notion that NB cells functionally resemble committed SA progenitors, while lacking specific stem cell program, provides a further understanding of NB biology and allows for better design of future therapeutic protocols for children affected by this malignancy.

## MATERIALS AND METHODS

### Mouse models

*Sox10-Cre* (B6; CBA-Tg (Sox10-cre)1Wdr/J; stock number: 025807), *LSL-tdTomato* (B6. Cg-Gt (ROSA)26Sortm14(CAG-tdTomato) Hze/J; stock number: 007914) and *Sox9-IRES-eGFP* (B6;129S4-Sox9tm1.1Tlu/J; stock number 030137) mouse strains were purchased from The Jackson Laboratory. *Dhh-Cre* mouse strain was received from Prof. Dies Meijer, also available at the Jackson Laboratory (FVB (Cg)-Tg (Dhh-cre)1Mejr/J; stock number: 012929). *Dbh-iCre* (Tg (Dbh-icre)1Gsc) (Parlato et al., 2007) mouse line was donated by Prof. Hermann Rohrer. *LSL-MYCN* (Gt (ROSA)26Sortm1(CAG-MYCN,-luc) Jhsc) was received from the lab of Prof. Johannes H. Schulte. *TH-MYCN* (Tg (Th-MYCN)41Waw) was acquired from the NCI mouse repository. *C57BL/6NRj* mouse strain was purchased from Janvier Labs. Nude mice (CAnN. Cg-Foxn1<nu>/Crl (BALB/c-nude)) were purchased from Charles River. All animal experiments were performed in agreement with the Swiss Law and were approved by the cantonal veterinary office of Zurich, Switzerland.

### Human tissue microarray and clinical tissue

Two neuroblastoma tissue microarrays were applied in this study. One slide containing 94 neuroblastoma cases was collected by the Children’s Hospital Zurich and the University Hospital of Zurich, another slide containing 27 neuroblastoma cases was commercially available and acquired from US Biomax. Primary and metastatic tumor sections from a single neuroblastoma patient, and two adrenal gland sections from fetus were provided by Dr. Peter Bode.

### Cell culture

Human neuroblastoma cell lines, CA2E, CHP-100, GI-ME-N, IGR-N-91, IMR-32, LAN-2, LAN-5, SH-EP, SK-N-DZ, and WSN were kindly provide by Dr. Annick Mühlethaler-Mottet. IMR-5, LAN-1, SH-SY5Y, and SK-N-AS were obtained from University Children’s Hospital Zurich. SK-N-BE (2) was purchased from American type culture collection (ATCC). All cell lines were culture in DMEM/F-12 medium (Thermo Fisher Scientific) supplemented with 10% fetal bovine serum (Dutscher), 1× L-glutamine (Thermo Fisher Scientific), 1× sodium pyruvate (Thermo Fisher Scientific), and 1× MEM non-essential amino acids (Thermo Fisher Scientific), and maintained in a humidified incubator at 37° C, 5% CO_2_. For immunocytochemistry, cells were seeded in μ-slide 8 well (ibidi) for culture.

### Genotyping and *in vivo* imaging

For genotyping, mice biopsies were lysed using lysis buffer (5M NaCl, 2M Tris pH 8-8.5, 0.5M EDTA, 20% SDS) and proteinase K at 55° C overnight. DNA was precipitated using isopropanol and centrifuged at 14000 rpm for 30 min. A washing step was performed using 70% ethanol, followed by centrifugation at 14000 rpm for 15 min. For PCR, the diluted DNA was added to a master mix with KAPA Taq ReadyMix (KAPA Biosystems), primers and performed according with KAPA Taq ReadyMix instructions. Primers are listed in supplementary information 4.

*In vivo* imaging system (IVIS) was used for tumor detection as previously described [4]. Briefly, animals were anesthetized and shaved using a waxing cream (Veet). Subsequently, animals were injected with 150 mg/kg of body weight with Luciferin (15 mg/ml in DPBS; 122799, Perkin Elmer) and imaged 10 min later.

### Analysis of public database of neuroblastoma samples and cell lines

SEQC/MAQC-III Consortium cohort (GSE49711) [48] contains gene expression profile from 498 neuroblastoma samples by RNA sequencing and microarray. Boeva et al. cohort (GSE90683) [37] contains RNA sequencing profiling of 25 neuroblastoma cell lines and 2 neural crest cell lines. Harenza et al. cohort (GSE89413) [49] includes transcriptomic profiling of 39 neuroblastoma cell lines performed by RNA sequencing. Kaplan-Meier curve of event free survival (EFS) and overall survival (OS) from SEQC/MAQC-III Consortium cohort were analyzed by R2: genomic analysis and visualization platform. Differentially gene expression with neuroblastoma clinical features and gene expression in Boeva et al. and Harenza et al cohorts were performed using R (version 3.5.0).

### Immunostaining

For paraffin embedded tissue, dissected mouse tissues were fixed in Histofix (Roth) at room temperature for 4 hours. Tissues were washed in PBS and dehydrated. Dehydrated tissues were then embedded in Paraffin and sections 5 μm thick were collected for histological analysis. Immunohistochemistry was performed following the instruction of VECTASTAIN ABC kit (Vector Laboratories). Paraffin-embedded tissue sections were deparaffinized and rehydrated. Antigen retrieval was done by incubation in citrate buffer at 110° C for 25 min. Slides were then incubated for 10 min in 3% H_2_O_2_ solution (Sigma Aldrich), washed in PBS and blocking buffer at room temperature was applied for 1 hour. Incubation with PBS-diluted primary antibodies was performed at 4° C overnight. The slides were washed 3 times in PBS and then incubated with HRP labeled polymer secondary and DAB substrate kit (Abcam) was used to visualize immunoreaction products. The sections were counterstained with hematoxylin (Biosystem) and mounted with EUKITT. For immunofluorescence, the tissue sections were blocked with 5% horse serum (Sigma Aldrich) and 0.1% triton X-100 (Sigma Aldrich) in PBS for one hour at room temperature after antigen retrieval. Slides were then incubated with primary antibody at 4° C overnight, washed in PBS and subsequently fluorescence conjugated secondary antibodies were applied for 1 hour at room temperature. At last, slides were mounted with fluorescence mounting medium (Dako) containing Hoechst (Life Technologies). For immunofluorescence on cryosections, slides were dried at room temperature and washed in PBS. Sections were permeabilized with 0.5% Triton X-100 for 5 minutes and were followed by procedure of immunofluorescence. For immunocytochemistry, cells were first fixed by Histofix for 10 minutes and then were followed by procedure of immunofluorescence.

### Plasmids and transfection

For stable overexpression of SOX9, human SOX9 expressing plasmid, pcDNA3-NFlag-SOX9fl (kindly provided by Prof. Michael Wegner), and vector control plasmid, pcDNA3-Flag-HA (kindly provided by Dr. Paolo Cinelli), were transfected into IMR-5, SH-SY5Y, SK-N-BE (2) neuroblastoma cell lines. Transfected cells were selected by G418 (IMR-5: 400 μg/ml, SH-SY5Y: 600 μg/ml, SK-N-BE (2): 800 μg/ml) to generated stable clones. For transient expression of SOX9, GFP-tagged SOX9-expressing plasmid, pCMV6-SOX9-GFP (Origene), and GFP-expressing plasmid, pSUPER-GFP (kindly provided by Prof. Michael Wegner), as control, were applied for transfection. Transfection was performed by JetPrime (Polyplus), following the producer’s instruction. For transient expression, cells were harvested at post-transfection 48 hours for immunostaining, western blotting and sorting.

Gene knockdown was performed by using lentivirus packaged short hairpin RNA (shRNA) construct (Sigma Aldrich). shRNA targeting SOX9 (pLKO.1 shSOX9_TRCN0000342824) and non-sense sequence (pLKO. shns), as negative control, were used for knockdown. To produce virus particle, HEK-293T/17 cells were co-transfected with packaging plasmid pPAX2, envelope plasmid pMD2G (both kindly provided by Dr. Christian Britschgi), and shRNA plasmids. Removed medium containing transfection mixture and replaced with fresh medium. After 24 hours, virus-containing medium was harvested and filtered through 0.45 μm filter, and frozen at –80° C. For infection, SK-N-AS cells were seeded one day prior to infection. On the second day, 8 ug/ml of polybrene and virus-containing medium were added to cells and incubated overnight. Cultured medium was removed and replaced by fresh medium. For generation of stable clone, 2 μg/ml of puromycin (Santa Cruz) was added for selection.

### Cell sorting

Cells transfected with pSUPER-GFP or pCMV6-SOX9-GFP (Origene) were trypsinized and resuspended in PBS for sorting. Cells were sorted by FACSAria III sorter (BD). Live cells were selected by staining with 1 μg/ml propidium iodine (PI) (Sigma) before sorting. Non-transfected cells, non-transfected cells with PI, and pSUPER-GFP transfected cell without PI served as control. Pre-gating for single and alive events was performed before setting the gate for GFP-positive events. Sorted cells were collected into lysis buffer from RNeasy Plus Micro kit (QIAGEN) for RNA isolation.

### RNA sequencing and analysis

Isolated RNA from 3 independent samples (*n =* 3 in each set of experiments) was submitted to Functional Genomic Center Zurich for sequencing. RNA sequencing was performed on Illumina Novaseq 6000 (Illumina). For following analysis, genes were selected based on p value lower than 0.05 and expression fold change is either above 2 or less than 0.5. Analysis of gene ontology was performed using Database for Annotation, Visualization and Integrated Discovery (DAVID) bioinformatics web tool, and gene set enrichment analysis (GSEA) was analyzed using GSEA software with signature gene set created based on published literature (Supplementary Information 3). The data are available under GSE133014.

### Protein extraction and Western blot

Harvested cell pellet was resuspended in RIPA lysis buffer (Thermo Fisher Scientific) containing protease inhibitor (Roche) and phosphatase inhibitor (Roche). Supernatant was collected by centrifugation at 14000 rpm for 30 min in 4° C. Protein concentration was determined by BCA protein assay (Thermo Fisher Scientific). Mixture of 30 μg protein and 4× Laemmli sample buffer (BioRad) was denatured at 95° C for 5 min. Protein mixture was loaded onto 4–20% TGX stain free precast gel (BioRad) for electrophoresis and transferred to PVDF membrane by Trans-Blot Turbo Blotting System (BioRad). The membrane was blocked with 5% w/v milk for 1 hour at room temperature and was incubated with TBS-T diluted primary antibodies overnight at 4° C. Membrane was next incubated with diluted HRP-conjugated secondary antibodies for 1 hour at room temperature. The blot was developed by adding Western Chemiluminescent HRP Substrate (BioRad) and the signal captured using Fusion FX Imaging System (Vilber).

### Migration and invasion assay

Cell migration assay was performed using 8mm pore size transwell (Falcon). Invasion assay was performed using Matrigel-coated invasion chamber (Corning). Cells were starved in 3% FBS starving medium for 48 hours prior to experiment. 5 × 10^5^ starved cells in serum free medium were seeded in the upper chamber and the lower chamber was filled with 0.6 ml of culture medium, followed by incubation at 37° C for 24 h. Cells were fixed in Histofix for 10 minutes and cells that remained in the upper side of the membrane were removed by cotton swab. Migrated and invaded cells at the bottom of the membrane were stained with Hoechst. Ten fields (20×) of each transwell were imaged and number of migrated and invaded cells in a field was calculated.

### Soft agar assay

Soft agar assay was performed by following instruction of CytoSelect 96-well cell transformation kit (Cell Biolab). Base agar matrix was prepared, 50 μl of agar matrix per well were loaded into a 96 well plate and allowed it to solidify. 5000 cells in 75 μl of cell suspension and agar matrix mixture were added to each well and allowed to solidify. 50 μl of culture medium was added and incubated for 7 days at 37° C, 5% CO_2_. After solubilizing the agar by adding solubilization solution, MTT solution was added and incubated 2 hours in dark at 37° C, 5% CO_2_. Detergent solution was added and incubated for 2 hours in dark at room temperature, followed by. Absorbance measurement at 570 nm by ELISA reader.

### Xenograft model

For heterotopic model, 1 × 10^6^ cells suspended in 200 μl serum free DMEM/F12 medium were injected subcutaneously in the back of nude mice. Tumor growth was monitored and measured twice per week. Long diameter (X)(mm) and short diameter (Y)(mm) of tumor were measured, and tumor volume was calculated by following equation: (X^*^Y [2])/2 (mm^3^) and mice were euthanized once the maximal tumor volume allowed was reached (X:10mm, Y:10mm). The experiments were performed in agreement with the Swiss Law and were approved by the cantonal veterinary office of Zurich, Switzerland.

### Retinoic acid induced differentiation model

For immunocytochemistry, 10000 cells were seeded in μ-slide 8 well (ibidi), and 3 × 10^5^ cells were seeded in 6 cm dish in culture medium for qPCR. On the second day, medium was replaced by 1% FBS culture medium plus 10 μM retinoic acid (RA)(Sigma Aldrich) and cultured for 48 hours. Medium was replaced again with RA contained 1% FBS medium, and cells were harvested for immunocytochemistry or qPCR after 48 hours of incubation.

### RNA extraction, reverse transcription PCR, and quantitative PCR

Total RNA was extracted by RNeasy Kit (QIAGEN) according to instructions and 1 μg RNA was used to synthesize cDNA in reverse transcription PCR (RT-PCR). RT-PCR was carried out using GoScript reverse transcriptase (Promega), and relative mRNA expression level was determined by quantitative PCR using Rotor-Gene SYBR green PCR kit (QIAGEN). Rotor-Gene Q real-time PCR system was used for qPCR assay with the following program: initial denaturation at 95°C for 10 minutes, 50 cycle of denaturation at 95°C for 10 seconds, annealing at 60°C for 10 seconds, and extension at 72° C for 30 seconds, final extension at 72°C for 10 minutes. Raw data was analyzed by ΔΔCT method and normalized to GAPDH.

### Tumor sphere formation assay

Desired numbers of cells were suspended in appropriate volume of tumor sphere medium DMEM/F-12 supplemented with 20 ng/ml epidermal growth factor (Peprotech), 10 ng/ml basic fibroblast growth factor (Peprotech), 0.4% bovine serum albumin (Roth), 1 × Insulin-Transferrin-Selenium (Gibco), and 1× B27 supplement (Gibco) to make cell concentration at 1 cell/μl. Two hundred cells were seeded in ultra-low attachment 96-well plates (Corning), and wells at the edge of plate were added with PBS. Plates were sealed with Parafilm to prevent evaporation of medium and incubated at 37° C, 5% CO_2_ for 10 days. Number of first passage spheres was counted using a phase-contrast microscope and spheres were then collected and trypsinized. Dissociated cells were used for second passage sphere formation assay.

### Quantification and statistical analysis

Results were obtained from at least three independent experiments. Mouse numbers are indicated in each figure. Data are shown as mean ± SEM, different group comparison test were performed as indicated in figures and all tests were applied unpaired and two-tailed method. Significance of Kaplan-Meier curves were analyzed by log-rank test. Statistical analysis was performed by using GraphPad Prism 8 software, R (version 3.5.0), and R2: genomic analysis and visualization platform.

### Data and code availability

RNA sequencing data have been deposited in NCBI Gene Expression Omnibus (GEO), accession number: GSE133014.

## SUPPLEMENTARY MATERIALS


