# Supplementary material for: Lineage-restricted sympathoadrenal progenitors confer neuroblastoma origin and its tumorigenicity
Source: Oncotarget. 2020 Jun 16;11(24):2357–71. doi: 10.18632/oncotarget.27636 (PMC7299536; doi:10.18632/oncotarget.27636)
Supplement: Supplementary file 1 [file oncotarget-11-2357-s001.pdf]

Lineage-restricted sympathoadrenal progenitors confer neuroblastoma origin and its tumorigenicity

SUPPLEMENTARY MATERIALS

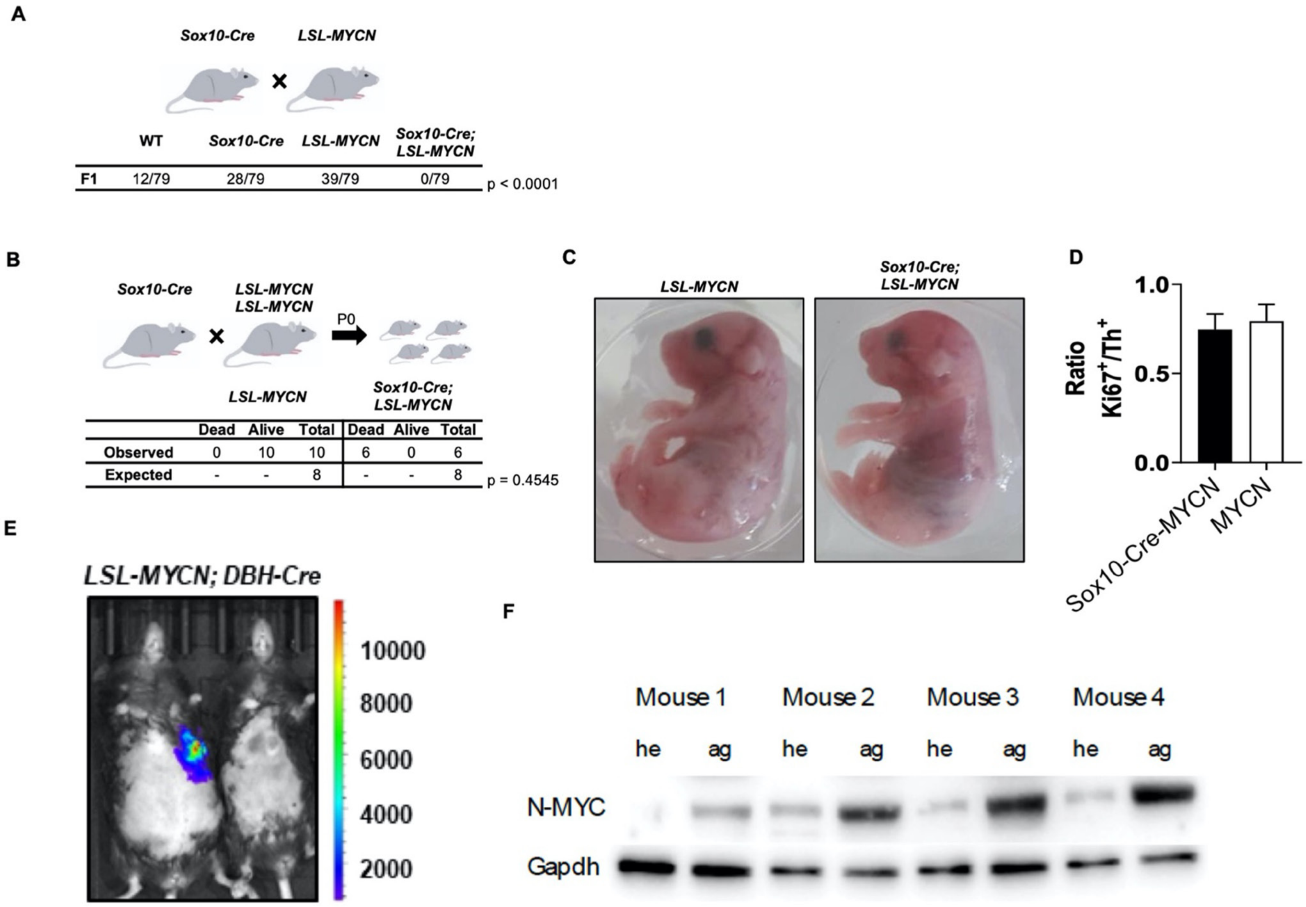

Supplementary Figure 1: (A) Genotyping results of F1 generation from *Sox10-Cre* and *LSL-MYCN* breeding. From all the F1 pups biopsied at post-natal day 20 *Sox10-MYCN* pups were never detected (Fisher's exact test, $p < 0.0001$). (**B**) Genotyping results from F1 pups found dead at postnatal day 0 and alive corresponding litter pups (Fisher's exact test, $p < 0.0001$). (**C**) Representative images of E18.5 pups from *Sox10-Cre* and *LSL-MYCN* breeding. (**D**) Bar graphs show ratio of Ki67 positive cells out of Th-positive cells from adrenal gland (AG) of *LSL-MYCN* (n = 3), and *Sox10-Cre; LSL-MYCN* (n = 3) E18.5 mouse embryo. Data are shown as mean ± SEM, two-tailed Mann-Whitney test was used. (**E**) *In vivo* imaging of *LSL-MYCN; Dbh-iCre* mice with (left) or without (right) tumor growth. (**F**) Western blot analysis of tissues from *LSL-MYCN; Dbh-iCre* mice showing the MYCN overexpression. He-heart, ag-adrenal gland.

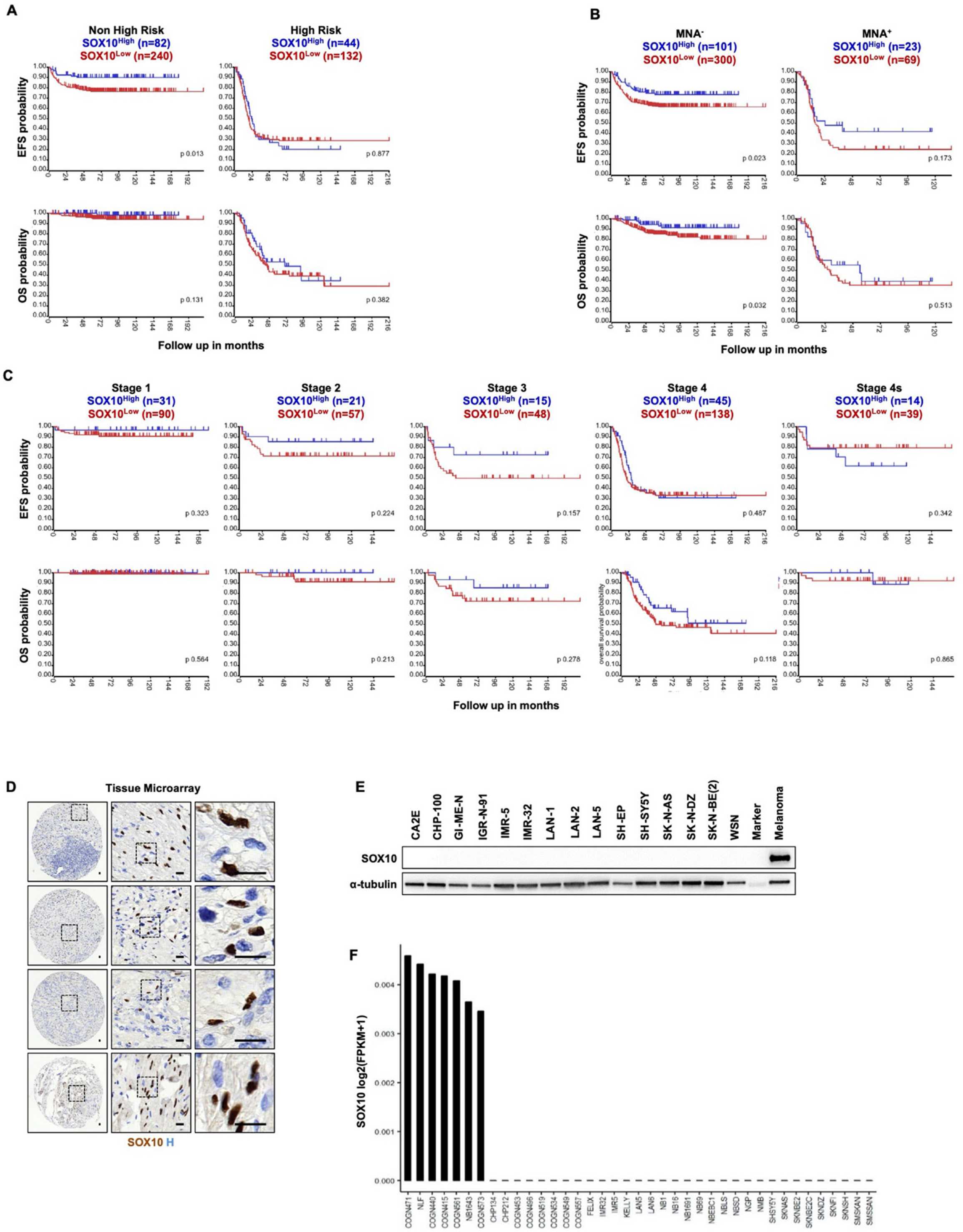

Supplementary Figure 2: (**A**) Kaplan–Meiercurve of event free survival (EFS) and overall survival (OS) of neuroblastoma patients with high or low SOX10 gene expression in non-high risk and high-risk group in cohort 1. Low SOX10 expression in non-high risk group indicates poor EFS survival rates. (**B**) Kaplan–Meiercurve of EFS and OS of neuroblastoma patients with high or low SOX10 gene expression in MNA$^-$ and MNA$^+$ patients in cohort 1. Low SOX10 expression shows worse EFS and OS survival rates in MNA$^-$ patients. MNA$^-$: MYCN non-amplification. MNA$^+$: MYCN amplification. (**C**) Kaplan–Meiercurve of EFS and OS of neuroblastoma patients with high or low SOX10 gene expression in INSS stages. Expression level was defined by using upper quartile cut-off. P value was determined by log-rank test. (**D**) Supplementary images of immunohistochemistry staining of SOX10 in stromal compartment of human tissue microarray. Scale bars: 20 µm (**E**) Western blotting analysis of SOX10 expression in extended neuroblastoma cell lines. Melanoma cell line, M111031, was used as positive control. α-tubulin was used as loading control. (**F**) SOX10 RNA level of 39 neuroblastoma cell lines from database 2 indicates extremely low expression of SOX10 in neuroblastoma cell lines.

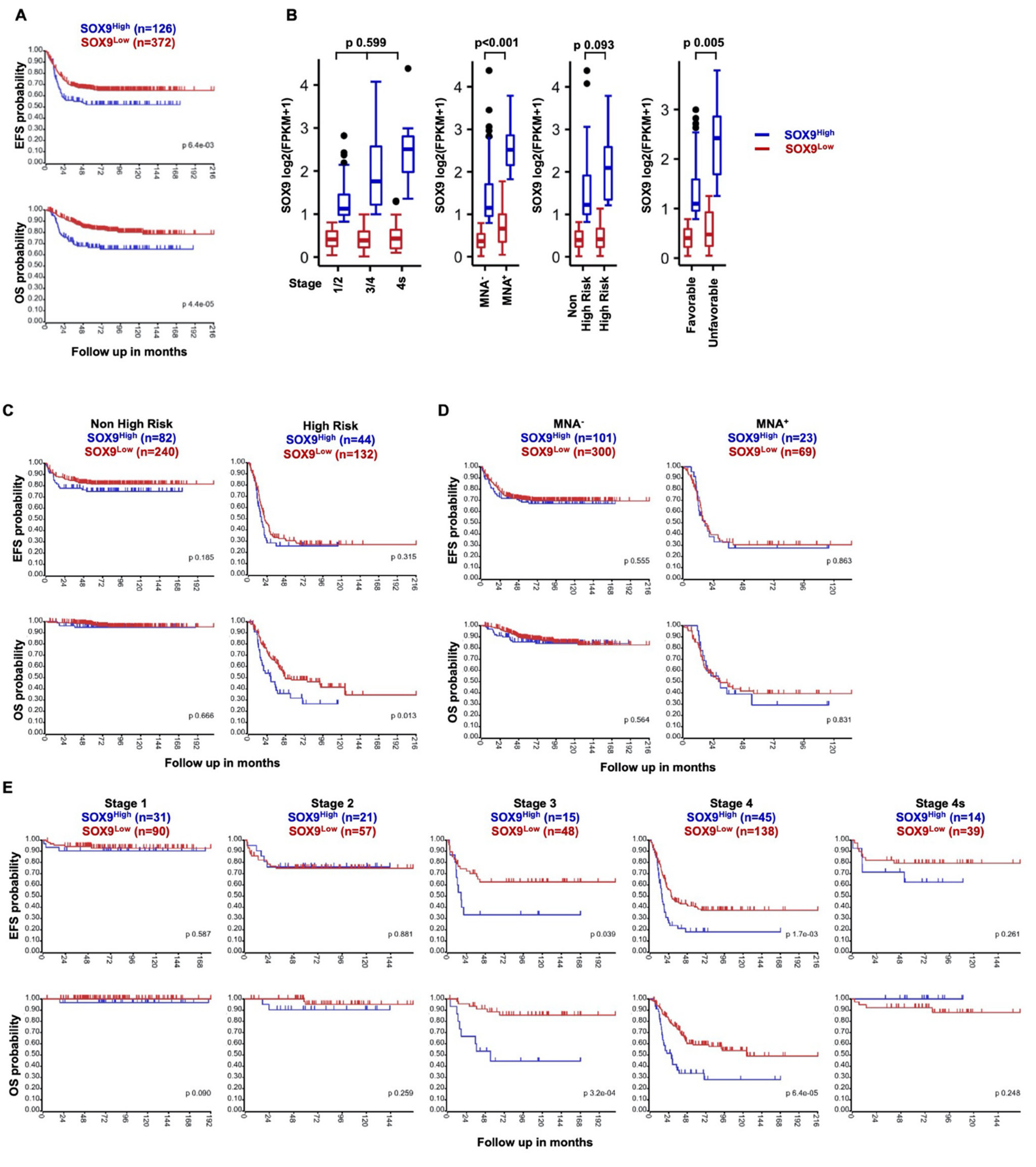

Supplementary Figure 3: (**A**) Kaplan–Meiercurve of event free survival (EFS) and overall survival (OS) of neuroblastoma patients with high and low SOX9 gene expression in cohort 1. (**B**) Boxplots graphs shows SOX9 expression in individual neuroblastoma clinical features including INSS stages (stage 1/2, $n = 199$; stage 3/4, $n = 246$; stage 4s, $n = 53$), MYCN status (MNA^-, $n = 401$; MNA^+, $n = 92$) risk group (non-high risk, $n = 322$, high risk, $n = 176$) and response to treatment (favorable, $n = 181$; unfavorable, $n = 91$) in cohort 1. For INSS stages, two-tailed Kruskal-Wallis test was used to calculate p value. For MYCN status, risk group, and response to treatment, two-tailed Mann-Whitney test was used to calculate p value. (**C**) Kaplan–Meier curve of EFS and OS of neuroblastoma patients with high or low SOX9 gene expression in non-high risk and high-risk group in cohort 1 (**D**), Kaplan–Meiercurve of EFS and OS of neuroblastoma patients with high or low SOX9 gene expression in MNA^- and MNA^+ patients in cohort 1. (**E**) Kaplan–Meiercurve of EFS and OS of neuroblastoma patients with high or low SOX9 gene expression in INSS stages. High SOX9 expression correlates to worse EFS and OS survival rates in stage3 and stage4 patients. (A, C–E) Expression level was defined by using upper quartile cut-off. *P* value was determined by log-rank test.

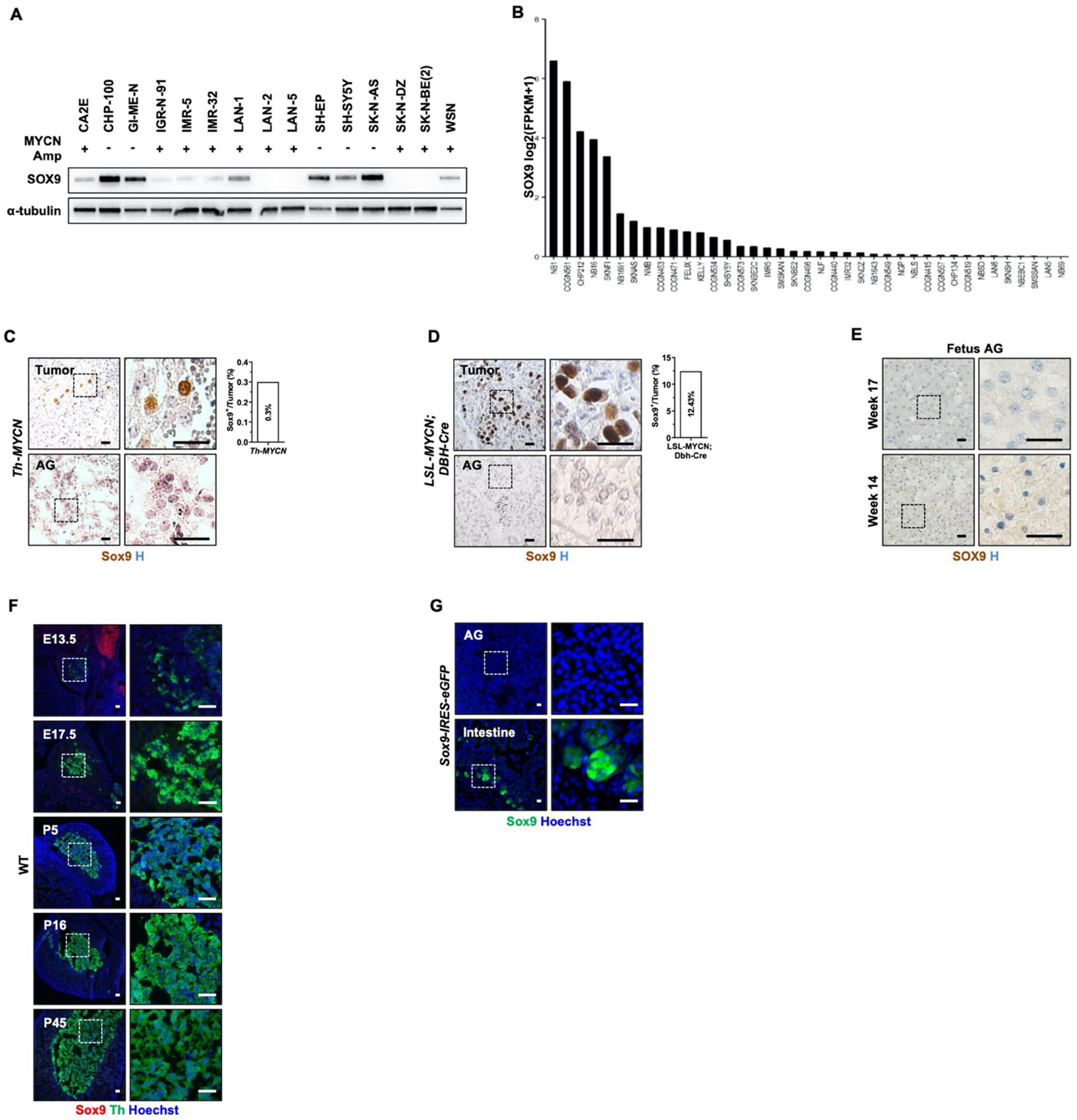

Supplementary Figure 4: (**A**) Western blot shows various expression level of SOX9 in human neuroblastoma cell lines. α-tubulin was used as loading control. (**B**) SOX9 RNA level of 39 neuroblastoma cell lines. (**C**) Immunohistochemistry staining of Sox9 in tumor and AG from *TH-MYCN* mouse model. Representative images show Sox9 was detected in a small population of tumor, but not in AG. Scale bar: 25 µm. (**D**) Sox9 staining in tumor and normal AG from *LSL-MYCN; Dbh-iCre* mouse model. Representative images show detection of SOX9 positive cells in tumor, but not in AG. Scale bar: 25 µm. (**E**) Immunohistochemistry shows negative staining of SOX9 in normal adrenal gland (AG) from week 17 and 14 fetuses. Scale bars: 25 µm. (**F**) Immunofluorescence staining of Sox9 and Th in AG from wild type mouse of indicated embryo (E) and postnatal (P) days. No Sox9 positive cells are detected in AG from wild type mouse. Scale bar: 50 µm. (**G**) GFP signal revealed no Sox9 positive cells in AG from Sox9-IRES-eGFP mouse. Intestine was used as positive control. Scale bar: 20 µm.

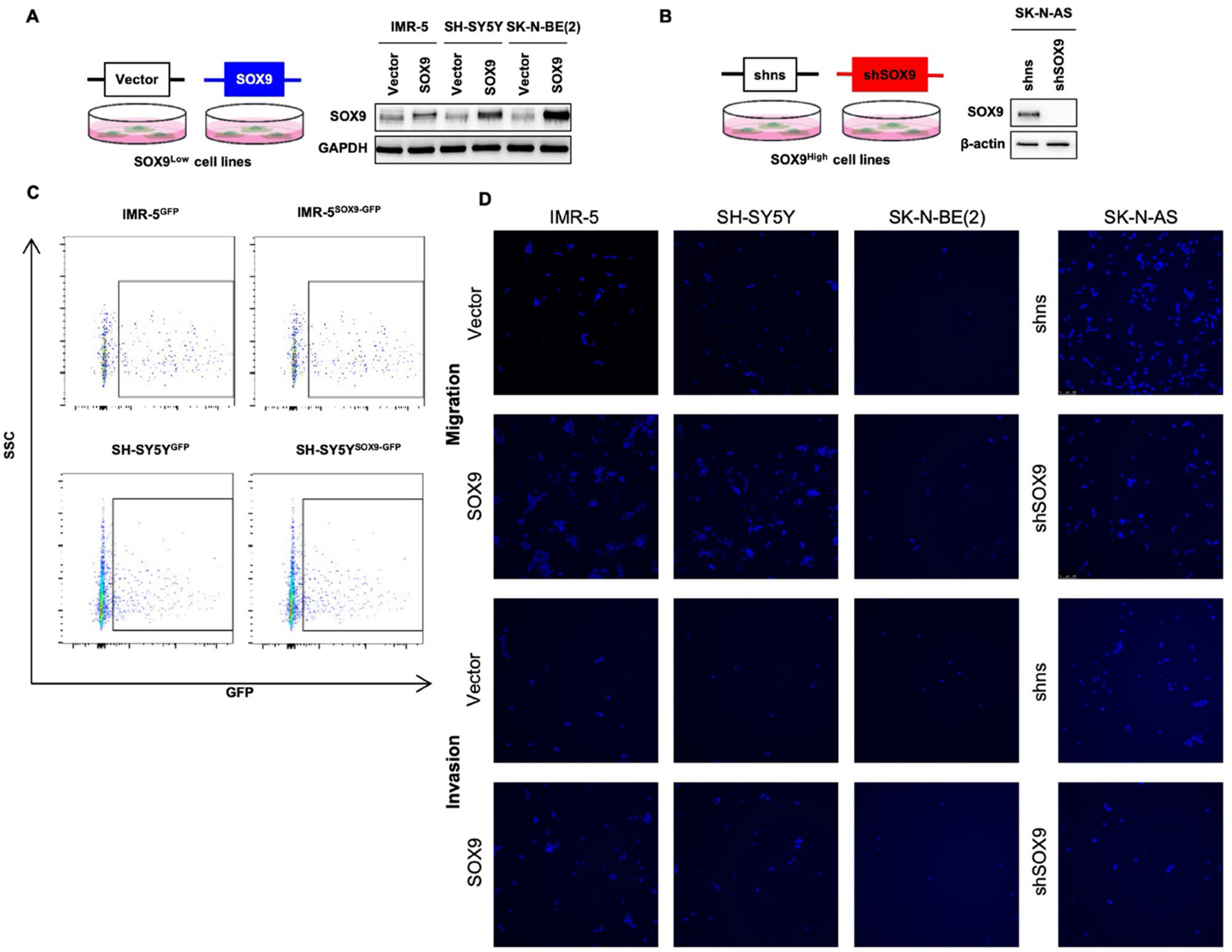

Supplementary Figure 5: (**A**) Schematic illustration of experimental strategy to overexpress SOX9 plasmid (SOX9) and vector control (vector) in low SOX9 expressing cell lines. Western blot shows SOX9 protein level upon overexpression in IMR-5, SH-SY5Y, and SK-N-BE(2) cells. (**B**) SOX9 shRNA (shSOX9) and non-sense shRNA (shns) in high SOX9 expressing cell line. Western blot shows knockdown in SK-N-AS. β-actin and GAPDH were used as loading control. (**C**) Graphs show results of FACS sorting GFP-positive cells from IMR-5 and SH-SY5Y cells transfected with GFP or GFP-tagged SOX9 expressing plasmid. (**D**) Representative images of migration and invasion assays are shown.

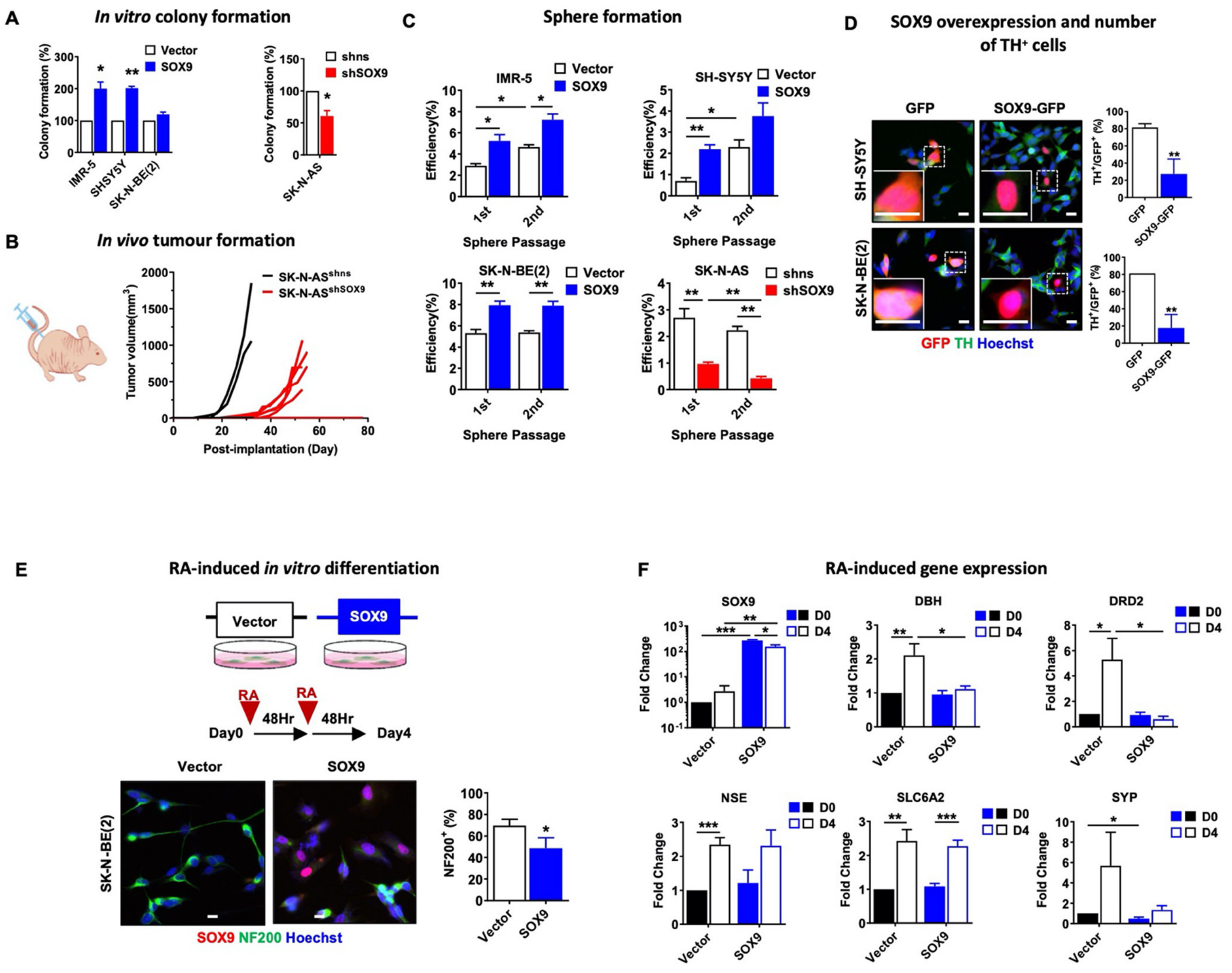

Supplementary Figure 6: (**A**) Soft agar assay of SOX9 overexpressing and knockdown cells. Graph shows fold change of absorbance value by normalizing to control groups of each cell lines. (**B**) Subcutaneous xenograft model by injecting SK-N-AS^{shns} or SK-N-AS^{shSOX9} cells. Graph presents growing curves of each injection. (**C**) Sphere formation assay of SOX9 overexpressed and knockdown cells. Percentage of sphere formation efficiency from first and second passage was measured by counting sphere numbers out of seeding cell numbers. Data were from three independent experiment and are shown as mean ± SEM. *P* value was determined by two-tailed *t*-test ($^{*}p < 0.05$, $^{**}p < 0.01$, $^{***}p < 0.001$). (**D**) Low SOX9 expressing cell lines were transfected with GFP or GFP-tagged SOX9. Cells were co-stained with GFP and sympathoadrenal lineage marker, TH, to determine differentiation status. Representative images are shown and graphs show percentage of TH positive cell out of GFP positive or SOX9 positive cells. Scale bar: 20 µm. (**E**) Transfected SK-N-BE (2) cells were treated with retinoic acid for 4 days, and were co-stained with SOX9 and neuronal marker, NF200. Representative images are shown and percentage of NF200 positive cells out of all cells was analyzed in multiple fields. Scale bar: 20 µm. (**F**) qPCR analysis of multiple neuron markers: *DBH*, *DRD2*, *NSE*, *SLC6A2*, and *SYP* in retinoic acid treated SH-SY5Y^{Vector} an SH-SY5Y^{SOX9} cell at day0 and day4. Fold change are calculated by comparing to SH-SY5Y^{Vector} at day0.
